# Sand mining in the Mekong Delta revisited - current scales of local sediment deficits

**DOI:** 10.1038/s41598-019-53804-z

**Published:** 2019-11-28

**Authors:** Christian Jordan, Jan Tiede, Oliver Lojek, Jan Visscher, Heiko Apel, Hong Quan Nguyen, Chau Nguyen Xuan Quang, Torsten Schlurmann

**Affiliations:** 1 Ludwig-Franzius-Institute for hydraulic, estuarine and coastal engineering, Leibniz University Hannover, Hannover, 30167 Germany; 2 GFZ - German Research Center for Geoscience, Section Hydrology, Potsdam, 14473 Germany; 3 Center of Water Management and Climate Change, Institute for Environment and Resources, Vietnam National University, Ho Chi Minh City, 700000 Vietnam; 4 Hydrology and Water Resources Research Group, Institute for Environment and Resources, Vietnam National University, Ho Chi Minh City, 700000 Vietnam

**Keywords:** Geomorphology, Environmental impact

## Abstract

The delta of the Mekong River in Vietnam has been heavily impacted by anthropogenic stresses in recent years, such as upstream dam construction and sand mining within the main and distributary channels, leading to riverbank and coastal erosion. Intensive bathymetric surveys, conducted within the Tien River branch during the dry and wet season 2018, reveal a high magnitude of sand mining activities. For the year 2018, an analysis of bathymetric maps and the local refilling processes leads to an estimated sand extraction volume of 4.64 $\pm$ 0.31 Mm${}^{3}$/yr in the study area, which covered around 20 km. Reported statistics of sand mining for all of the Mekong’s channels within the delta, which have a cumulative length of several hundred kilometres, are 17.77 Mm${}^{3}$/yr for this period. Results from this study highlight that these statistics are likely too conservative. It is also shown that natural sediment supplies from upper reaches of the Mekong are insufficient to compensate for the loss of extracted bed aggregates, illustrating the non-sustainable nature of the local sand mining practices.

## Introduction

Anthropogenic stresses, such as groundwater extraction, river training, construction and operation of hydropower infrastructure as well as sand mining play an important role in the future evolution of the world’s largest river deltas^1–3^. These human-induced processes cause altered freshwater and sediment discharge, rendering deltas prone to sea-level rise driven by climate change^4^. A prime example for these sketched developments is the delta of the Mekong River in the south of Vietnam, where the delta aggradation rate of 0.3 to 1.8 mm/yr^5^ is exceeded by land subsidence rates of several centimetres per year^6,7^ and rates of absolute sea-level rise of 2 to 4 mm/yr^8^. This imbalance is particularly concerning, because recent findings indicate that the mean delta elevation is only 0.8 m above the sea-level^9^, significantly lower than previously assumed. The Vietnamese Mekong Delta (VMD) is the third largest river delta worldwide with an area of around 41,000 km^2^ and a population of about 18 Mio. people^10^. The VMD is also known as the ‘rice bowl’ of Vietnam, since it provides over 50% of the country’s food production^10^.

Extraction of groundwater leads to substantial land subsidence within the VMD, directly imposing increasing risks of coastal floods^6,7^ and salinity intrusion^11^. The construction of dykes, sluice gates and pumps, which control the inundation of rice fields^5,12^, shifts the hazard of fluvial flood events downstream^13^. In recent years, the effect of upstream dams on the downstream riparian countries has gained particular attention^14^. The construction and operation of these dams alter the monsoon-driven hydrological regime^15,16^, concurrently leading to a shortage of sediment supplies for the sustainment of the delta region^17,18^ and hindering fish migration^19^. As of 2017, 44 hydropower dams were under construction within the Mekong Basin, with 212 additional projects being commissioned^20^. The illustrated developments will most likely only aggravate as soon as these dams become operational. At the start of the century, around 35 to 45 Mt of sediment were trapped per year due to dam operation^21^. In case that all proposed dams are being built, the sediment trapping could increase up to between 95 and 100 Mt/yr^21^, reflecting around 70% of the river’s sediment discharge. Some studies even assume a reduction in sediment supply to the sea by more than 95% for this case^17,18^. Recently, additional research focus has also shifted towards the non-sustainable mining of riverbed aggregates from the Mekong’s channels and its effects on the local sediment transport and river morphology^22,23^. Existing estimates for the sand extraction within the VMD are of the order of 7.7 to $20\ {Mm}^{3}$/yr^22,23^. The growing demand for aggregates mainly stems from construction and land reclamation projects. Drivers for these projects are population and economic growth as well as urbanisation. Globally, sand and gravel represent the second highest volume of raw material extracted and consumed by mankind, second only to water resources^24,25^. The mining of riverbed sediments on large scale cannot be fulfilled without impacts on the local environment. Effects on hydrology, sediment transport, biodiversity and groundwater levels are well documented^24–26^. River deepening due to sand mining can also trigger the import of mud into deltaic channels and increase the salinity intrusion^27^. This multitude of possible consequences has led to the control of mining activities or even the prohibition thereof in many countries^22,26^. The cumulative sediment starvation, which results from dam construction, sand mining and a shifting of tropical-cyclone activity^28^, has caused progressing bank erosion^29,30^, coastlines in recession^31–33^ and a loss of mangrove forests^34^.

In order to provide a robust estimate of sand mining activities based on direct river bed observation, this study focuses on the morphological evolution of sand mining sites. The selected sites were monitored systematically during the dry and wet season in 2018. In the course of the study, vessel-based highly-resolved bathymetric surveys were conducted along a 20 km stretch of the Tien River branch within the VMD. In addition, a unique set-up of sensors was used to gain insight into the local hydrodynamic and sediment transport processes over one seasonal cycle. The here presented results provide a deeper understanding of the relationships between local sand mining activities, sediment supplies and river morphology. Furthermore, the high spatial resolution of bathymetric datasets enables an accurate estimation of sand volumes extracted within the study area. Compared to reported statistics for the year 2018, discrepancies become apparent. Due to apparently informal mining activities, these statistics are likely too conservative. The combination of the different datasets also permits a critical assessment of resulting sediment deficits and plausible effects of future morphological developments within the VMD.

## Hydro-, Morpho- and Sedimentological Regime of the Mekong River and Study Area

The Mekong River has a length of about 4,900 km and traverses six countries (China, Myanmar, Thailand, Laos, Cambodia and Vietnam) on its way from the source on the Tibetan Plateau to the river mouth at the South China Sea. Within the delta region, the Mekong is divided into two main branches, the Tien River (‘Mekong proper’) and Hau River (‘Bassac’), which bifurcate into a total of eight major distributaries on their way to the coast. Near the Vietnamese border to Cambodia, the Tien River and Hau River are linked through the Vam Nao channel. Upstream of this junction, the Tien River carries around three quarters of the Mekong’s discharge volume, while it is almost equally split between both branches downstream from it^5^. The hydrological regime of the Mekong is typically characterised by a dry season (November-May) and a wet season (June-October). The Western North-Pacific and Indian monsoon lead to heavy precipitation within the Mekong Basin during the wet season, accounting for around 80% of its yearly discharge^35^. The mean annual discharge is about 13,200 m^3^/s at Kratie (Cambodia), upstream from the delta region^35^. The mean monthly discharge can vary between 1,600 to 37,000 m^3^/s, following the dry and wet season^35^. Estimates for the total yearly sediment discharge to the South China Sea vary from 40 to 160 Mt/yr^36–38^. Sand, transported as suspended load, contributes 6.5 $\pm$ 1.6 Mt/yr to the total sediment transport^39^. Available data of bed load transport rates at Kratie and for the Hau River branch indicate that sand, transported as bed load, accounts for around 1 to 3% of the local sediment transport^39,40^.

**Figure 1:**
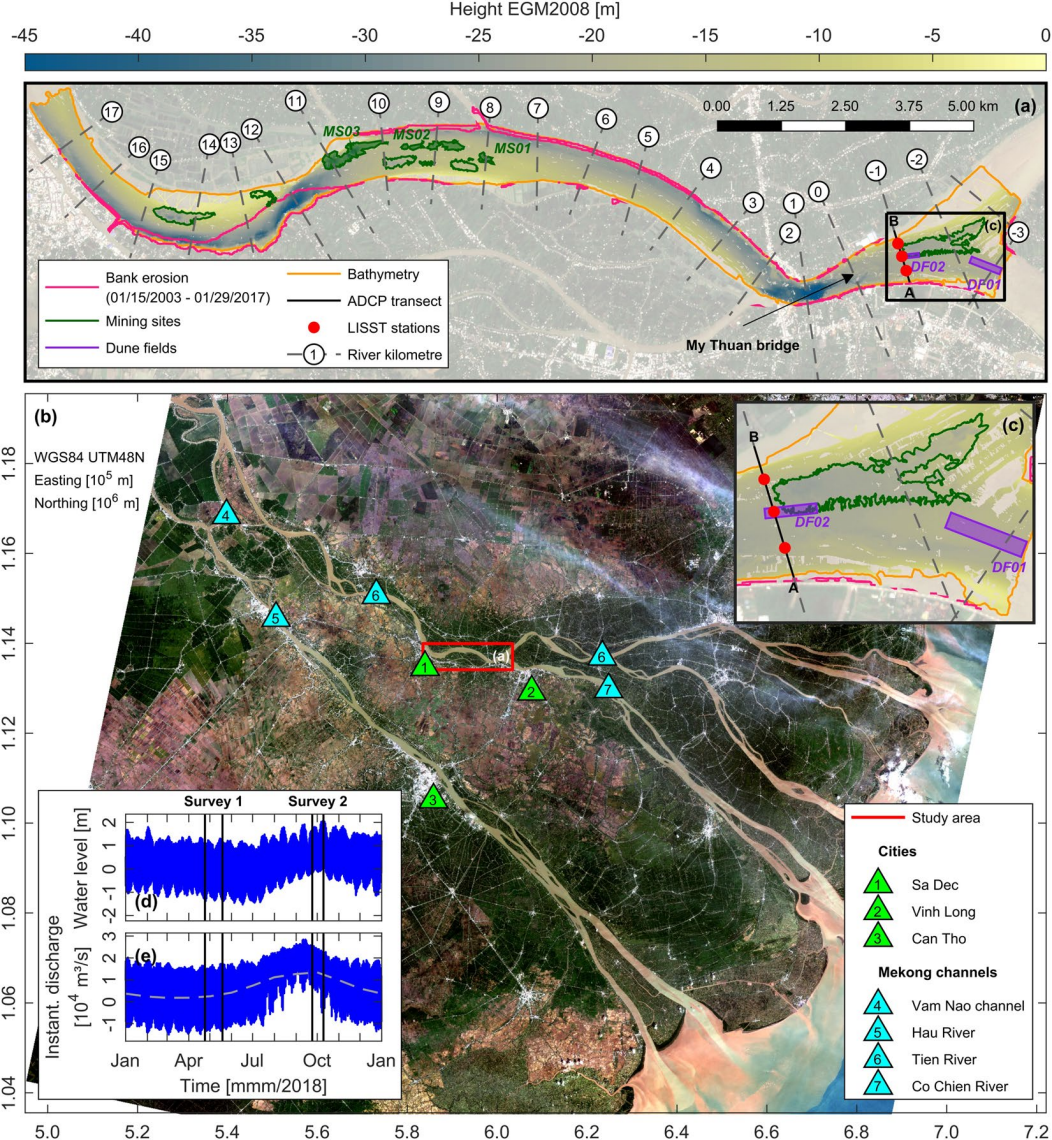
Map of the VMD and study area with bathymetry and locations of investigated mining sites, dune fields and measurement sites. (**a**) Map of the study area. Areas with bank erosion were extracted from historical Landsat-7 and 8 imagery using the $MNDWI$^59^. Sentinel-2 satellite data from October 28, 2018, shortly after the completion of the second survey, was used as background image. (**b**) Map of the VMD based on Landsat-8 imagery from October 31, 2018. (**c**) Detailed view of the bifurcation spot between the Tien River and Co Chien River. (**d**) Water level at station My Thuan (Vietnam) for the year 2018. (**e**) Instantaneous discharge at station My Thuan for the year 2018. Grey dashed line indicates the mean hydrograph for the years 2009–2016^48^. Raw data for water level and discharge was provided by the SRHMC. Landsat-7, Landsat-8 and Sentinel-2 (ESA) images courtesy of the U.S. Geological Survey (USGS), downloaded from the USGS Earth Resources Observation and Science (EROS) Center (https://earthexplorer.usgs.gov/). Copernicus Sentinel data 2018, processed by ESA. Illustrations (**a**–**e**) were generated using Matlab 2018a (http://mathworks.com).

The VMD study site is located on a riverine stretch of the Tien River branch. This section, which is located between the city of Sa Dec and the bifurcation spot of the Tien River and the Co Chien River near Vinh Long, covers around 20 km (Fig. 1). In order to capture seasonal variations caused by the dry and wet season, vessel-based surveys were carried out from April 24 to May 19 and September 24 to October 10, 2018, respectively. A unique set-up of sensors was applied to collect data and evaluate key parameters of the local hydro-, morpho- and sedimentological processes. In-situ acquired data included bathymetry, flow velocities, discharges, water levels, total suspended solids (TSS) and particle-size distributions. Bathymetric surveys were conducted via a multibeam echosounder (MBES), while an acoustic Doppler current profiler (ADCP) measured flow velocities over water depth to derive volumetric discharges. Volumetric sediment concentrations and particle-sizes within the water column were recorded via a laser in-situ scattering and transmissiometry (LISST) probe at multiple stations along selected ADCP transects (see A-B in Fig. 1a,c). Samples of water and bed sediment were taken to calibrate deployed sensors and to gather additional information about local sediment properties. These datasets were later complemented by continuous measurements of water levels and flow velocities for the whole year 2018 at My Thuan (Vietnam), provided by the Southern Regional Hydro-Meteorological Centre (SRHMC).

Within the study area, tidal currents from the South China Sea interact with riverine discharge, leading to a bidirectional flow regime during the dry season. During the wet season, currents are almost exclusively directed towards the ocean, induced by the monsoon-driven amplification of riverine discharge from the upstream basin. Instantaneous discharge for the year 2018 ranged between -15,000 to 28,400 m^3^/s (Fig. 1e and Supplementary Table S3), with negative values indicating upstream directed flow due to tidal influence. Riverine discharge, which was calculated by averaging the instantaneous discharge over a full tidal cycle, varied between around 1,000 to 21,000 m^3^/s (Supplementary Fig. S5). Tides within the study area are characterised by a mixed, mainly semidiurnal regime. Tidal range exceeded 1.7 m during the dry season and was smaller than 1.4 m during the wet season, accompanied by a steady increase in the mean water level (Fig. 1d and Supplementary Table S4). Bed aggregates within the area mainly consist of fine to medium sands (Supplementary Fig. S2 and ref. 41). An increase in median grain-size (${D}_{50}$) can be observed near deep scour holes, while mud content increases adjacent to shallow riverbanks.

**Figure 2:**
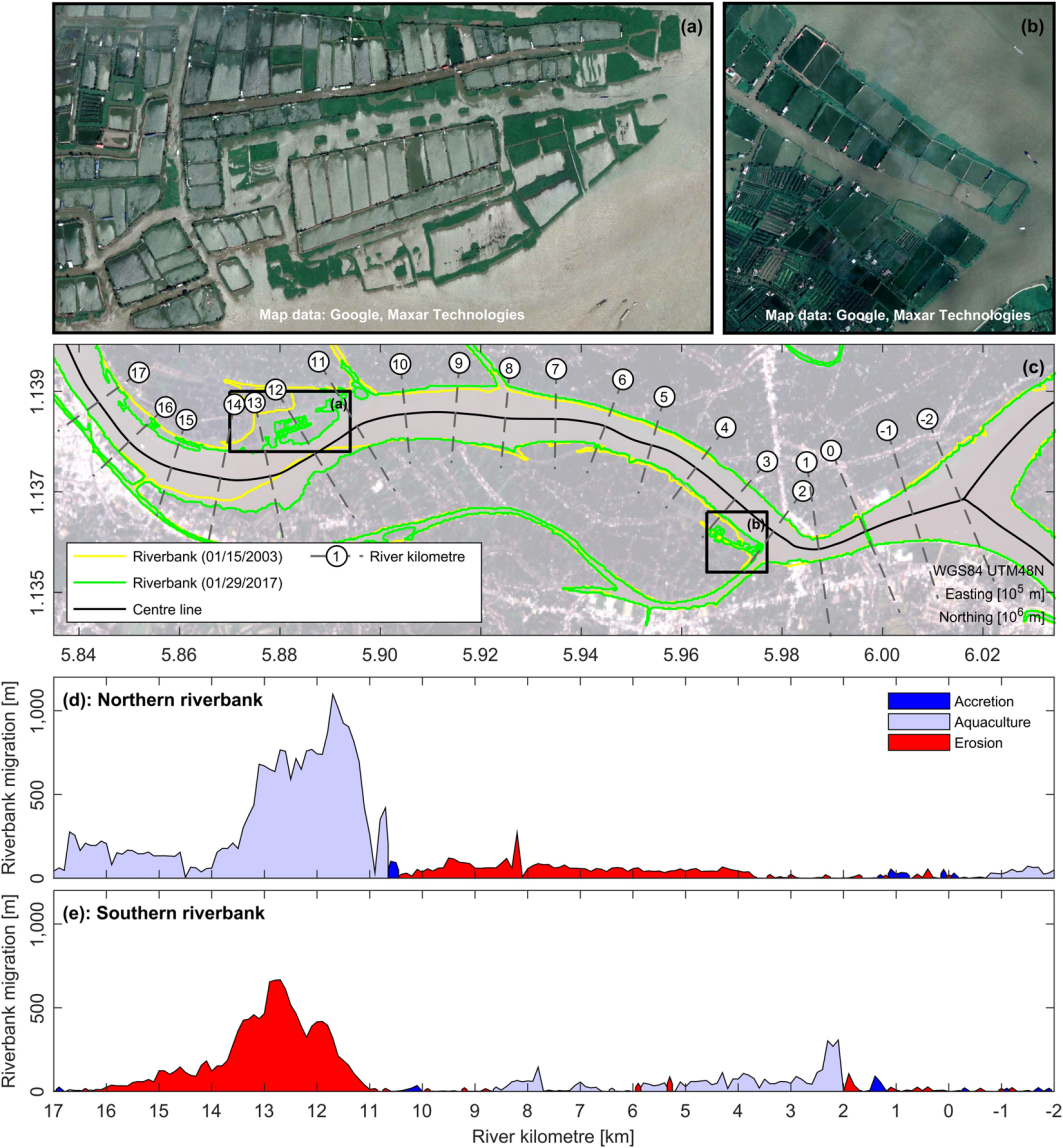
Changes in riverbank position within the study area between January 2003 and January 2017. (**a**,**b**) Aquaculture infrastructure built between January 2003 and January 2017. (**c**) Map with locations of riverbanks for January 15, 2003, and January 29, 2017. Riverbanks were extracted from historical Landsat-7 and 8 imagery using the $MNDWI$^59^. Landsat-8 image from January 29, 2017, was used as background image. (**d**) Accretion and erosion along the northern riverbank (RKM $-2$ to 17) between January 2003 and January 2017. (**e**) Accretion and erosion along the southern riverbank (RKM $-2$ to 17) between January 2003 and January 2017. Riverbank migration, i.e. accretion and erosion, was calculated along lines orthogonal to the centre line of the river. Accretion corresponding to locations of aquaculture infrastructure was highlighted separately. Satellite images shown in (**a** and **b**) were provided by Google Earth (https://earth.google.com/web), based on data from Maxar Technologies for December 22, 2017. Landsat-7 and Landsat-8 images courtesy of the USGS, downloaded from the USGS EROS Center (https://earthexplorer.usgs.gov/). Illustrations (**a**–**e**) were generated using Matlab 2018a (http://mathworks.com).

Maximum water depths within the study area locally exceed 50 m in the meandering river bends, where deep scour holes have formed (Fig. 1a). Underwater slopes in the proximity of the riverbanks can clearly surpass angles of 50° regularly leading to bank failure (see Supplementary Fig. S6e) due to excessive shear stresses. Analysis of exemplary Landsat satellite images reveals that massive bank erosion occurred within the study area during the last decade, i.e. 2003–2017 (Figs. 1a and 2c-e). In order to map morphological changes, a virtual river kilometre marker (RKM) was introduced along the river’s centre line (see Figs. 1a and 2c), with its origin at the location of the My Thuan bridge. Morphological activity leading to bank failure was particularly prominent between RKM 11 to 16, where the river channel shifted southward and narrowed. In total, more than 1.86 ${km}^{2}$ of land was lost to erosion between January 2003 and January 2017. Over the same period, the majority of the detected accretion of 2.47 ${km}^{2}$ corresponds to the expansion of aquaculture infrastructure (Fig. 2a,b). Thus, this accretion must rather be attributed to regional land-use changes and anthropogenic land accretion than a natural evolution of morphological processes. Furthermore, it seems likely that the observed pronounced meandering of the river, which led to the massive bank erosion, was triggered or at least accelerated by sediment trapping due to dams and local sand mining activities. In order to prevent continuous erosion of riverbanks and underwater slopes, groyne fields perpendicular to the river flow have been installed at multiple locations in recent years (Supplementary Fig. S6d). Furthermore, revetments have been created to protect erosion-prone urbanised areas (Supplementary Fig. S6c).

## Estimates of Local Sediment Transport for the 2018 Dry and Wet Season

Volumetric sediment concentrations and flow velocities were measured by means of LISST and ADCP at selected stations along transect A-B (see Fig. 1a,c) during both conducted surveys. Volumetric sediment concentrations were later calibrated against TSS from water samples (Supplementary Fig. S7). The total suspended transport for transect A-B was extrapolated from the product of TSS and flow velocity, measured at the stations. During the dry season survey, suspended transport was directed upstream during tidal high-water (flood) and downstream during tidal low-water (ebb). Maximum measured suspended transport rates were of the order of around 600 kg/s (Supplementary Fig. S7). During the wet season survey, suspended transport was solely directed towards the ocean, with a maximum magnitude of about 2,450 kg/s (Supplementary Fig. S7).

**Figure 3:**
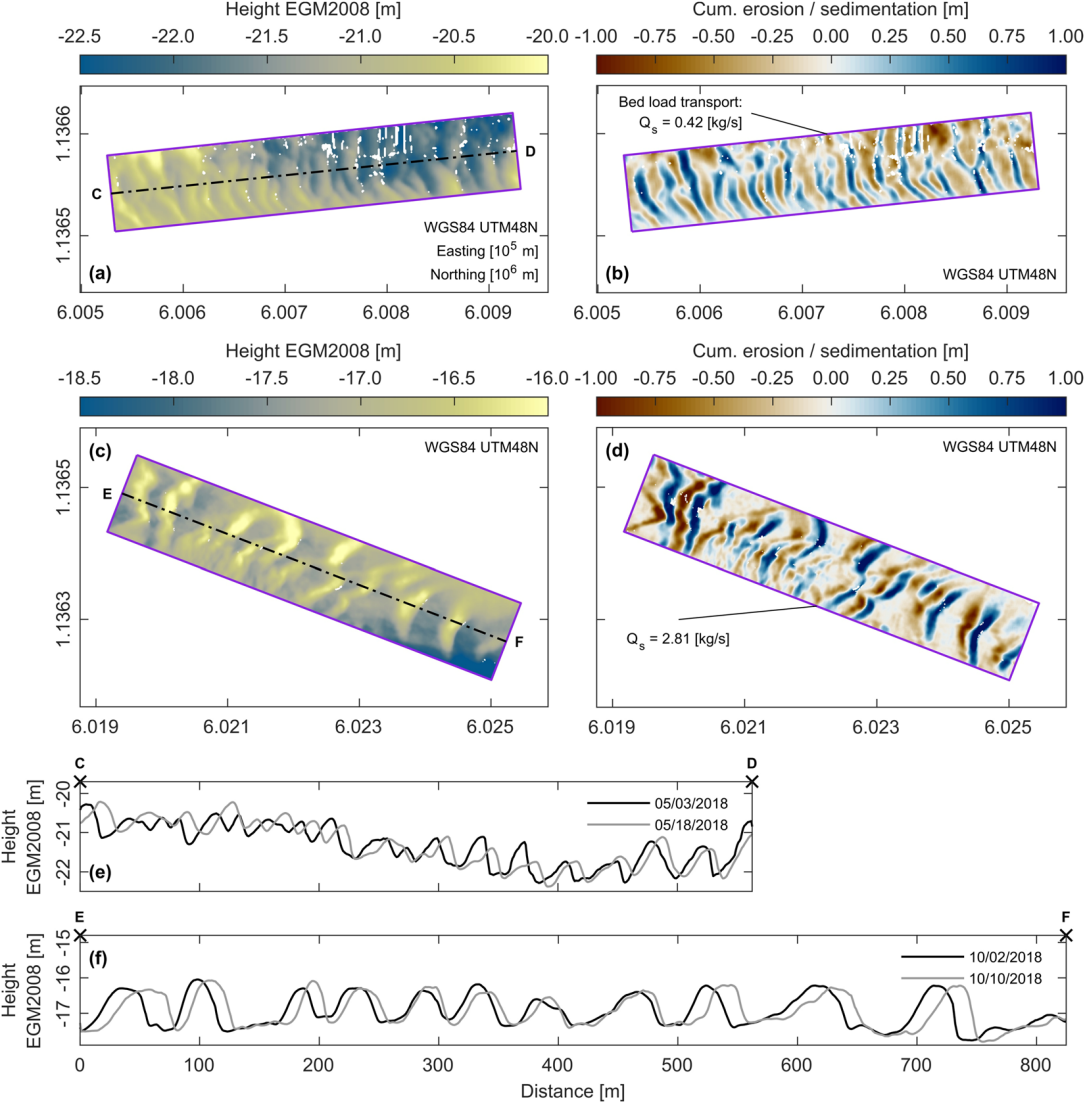
Bathymetric changes and associated bed load transport rates within dune fields *DF01* (wet season) and *DF02* (dry season). (**a**) Bathymetric map of dune field *DF02* on May 3, 2018. (**b**) Cumulative erosion (red) and sedimentation (blue) within dune field *DF02* from May 3 to 18, 2018. (**c**) Bathymetric map of dune field *DF01* on October 2, 2018. (**d**) Cumulative erosion (red) and sedimentation (blue) within dune field *DF01* from October 2 to 10, 2018. (**e**) Migration of dunes along cross-section C-D (see panel (**a**)) during the dry season 2018. (**f**) Migration of dunes along cross-section E-F (see panel (**c**)) during the wet season 2018. Note the different ranges of colourbars. Illustrations (**a**–**f**) were generated using Matlab 2018a (http://mathworks.com).

Bed load transport rates within the study area were quantified indirectly based on data acquisition and subsequent analysis of mobile bedforms along the riverbed. A characteristic dune field near the bifurcation of the Tien River with the Co Chien River was surveyed extensively during both surveys and selected to be analysed in full detail. During the dry season, the bedforms covered a section of the Tien River upstream of the bifurcation (see *DF02* in Fig. 1c) and extended into the Co Chien River (see *DF01* in Fig. 1c). During the wet season, the bedforms within the Tien River section were seemingly washed out, while only the ones within the Co Chien River section remained. According to lengths of 30.24 m and 67.20 m (see Supplementary Table S5), the assessed bedforms could be classified as large dunes^42^. Dune asymmetry (i.e. the ratio of a dune’s lee side length to its overall length), which is recognised as a robust proxy for dune migration^43^, showed a downstream directed migration within all acquired datasets (see Supplementary Table S5). The bedform translation method^44^, which analyses the migration of bedforms along the riverbed, was used to estimate bed load transport rates. Estimates of bed load transport for the dry season concentrated on dune field *DF02*, which was monitored at the beginning and end of the first survey. Resulting bed load transport rates for dune field *DF02* were of the order of 0.42 kg/s (Fig. 3b,e). During the wet season, bathymetric surveys concentrated on dune field *DF01*, which was monitored periodically in intervals of approximately 48 hours. The results showed that local bed load transport rates can vary considerably over the course of half a spring-neap cycle (Supplementary Fig. S8). The average bed load transport rate, based on the first and last bathymetric map of the wet season survey, amounts to 2.81 kg/s (Fig. 3d,f). Maximum observed rates on shorter time scales showed peak transport rates of the order of 9.61 kg/s (see Supplementary Fig. S8).

## Sand Mining Volumes and Refilling Processes at Mining Sites

**Figure 4:**
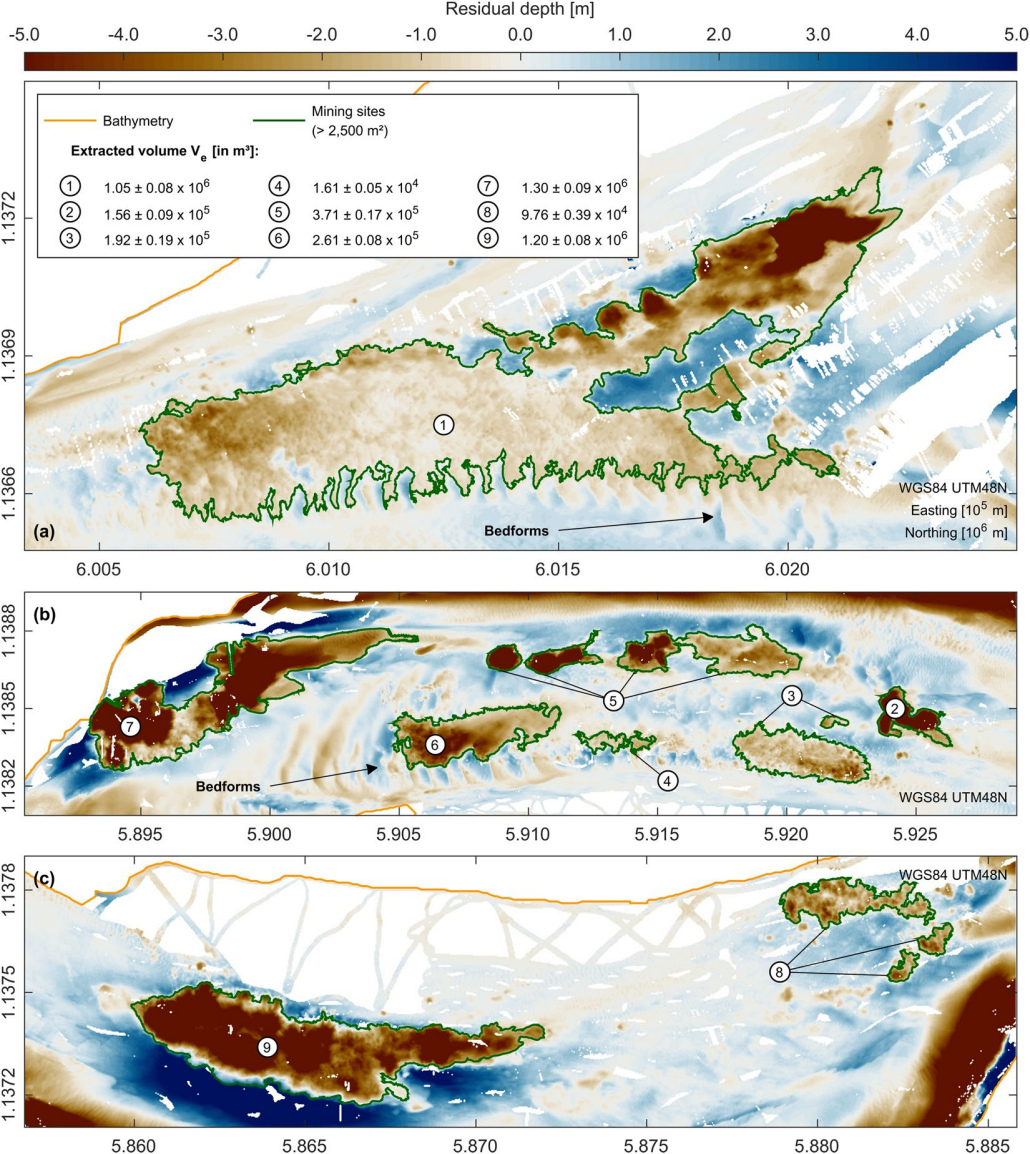
Detailed map of sand mining sites detected during the dry season 2018 and associated extraction volumes. (**a**) Residual bathymetry with mining sites between RKM $-3$ to $-1$. (**b**) Residual bathymetry with mining sites between RKM 8 to 11. (**c**) Residual bathymetry with mining sites between RKM 12 to 15. Red colour indicates areas below the median, while blue colour indicates areas above the median. Shown residual bathymetries are based on an exemplary filter width of 500 m. Note that this filter width does not necessarily reflect the optimised diameter for each mining site according to the ORS algorithm^52^. Illustrations (**a**–**c**) were generated using Matlab 2018a (http://mathworks.com).

**Figure 5:**
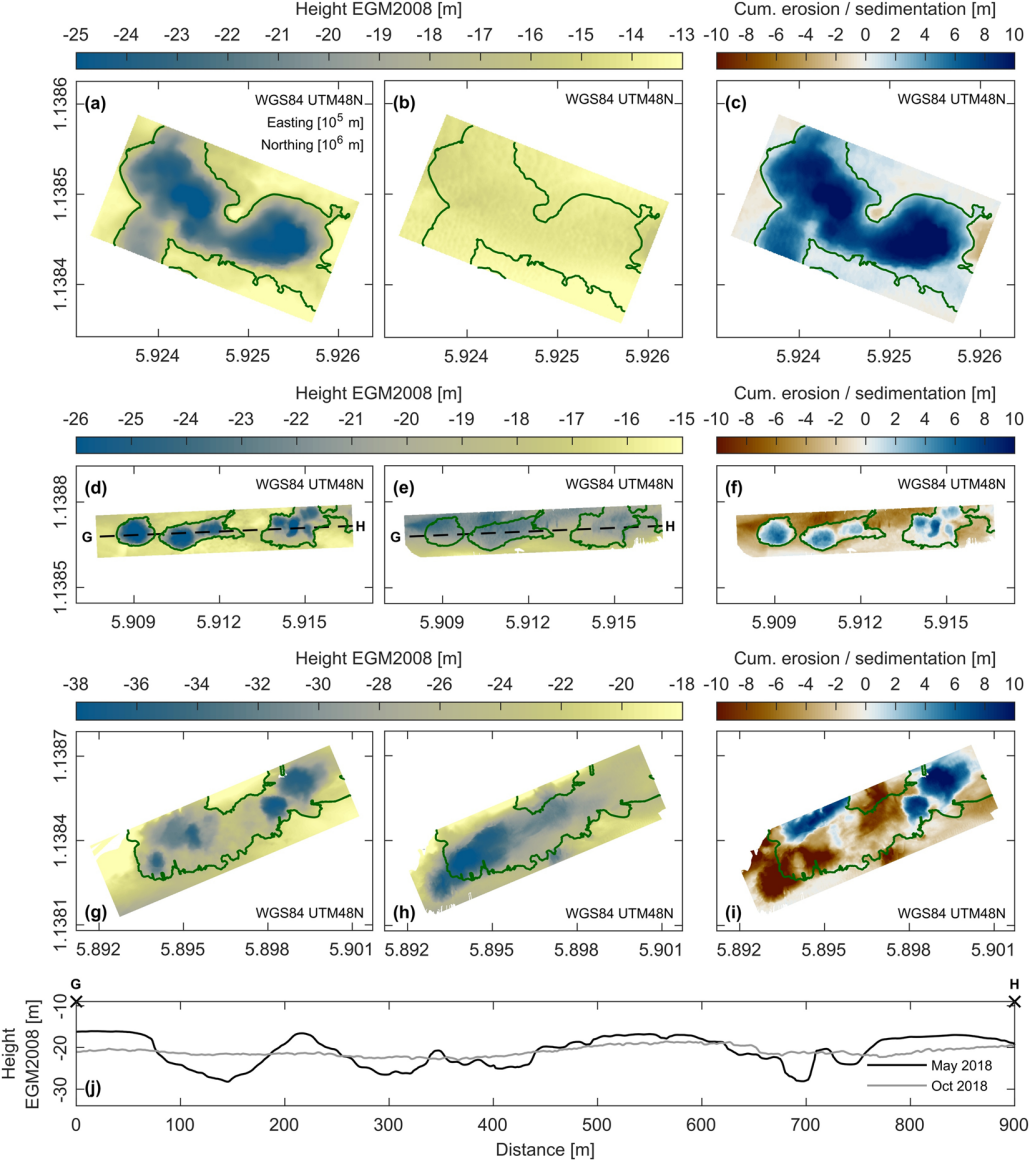
Morphological evolution of sand mining sites within study area. (**a**) Mining site *MS01* in early May 2018. (**b**) Mining site *MS01* in early October 2018. (**c**) Cumulative erosion (red) and sedimentation (blue) at mining site *MS01* from May to October 2018. (**d**) Mining site *MS02* in early May 2018. (**e**) Mining site *MS02* in early October 2018. (**f**) Cumulative erosion (red) and sedimentation (blue) at mining site *MS02* from May to October 2018. (**g**) Mining site *MS03* in early May 2018. (**h**) Mining site *MS03* in early October 2018. (**i**) Cumulative erosion (red) and sedimentation (blue) at mining site *MS03* from May to October 2018. (**j**) Evolution of mining site *MS02* along transect G-H (see panels (**d** and **e**)) from May to October 2018. Note the different ranges of colourbars. Illustrations (**a**–**j**) were generated using Matlab 2018a (http://mathworks.com).

Sand mining activities, i.e. the exploitation of sand depots from the riverbed to meet the demands of the local construction industry, are known to be substantial within the study area^22,23^. Single extraction sites can extend over several kilometres, with deep pits of over 17 m incised into the surrounding bathymetry (Figs. 4 and 5). During the dry season survey, all mining sites within the study area were mapped. The total sand mining volume of these locations was estimated based on residual bathymetries, i.e. the separation of smaller scale bathymetric features from the surrounding regional bathymetry. The resulting volume amounts to around 4.64 $\pm$ 0.31 ${Mm}^{3}$, with individual mining sites contributing up to 1.30 $\pm$ 0.09 ${Mm}^{3}$ (see Fig. 4). Applying a bulk density of 1.86 t/${m}^{3}$ (30% porosity and 2,650 kg/${m}^{3}$ grain density) this corresponds to a total excavated mass of 8.63 $\pm$ 0.58 Mt from the bed of the Tien River section. This volume and mass were also assumed to be a lower limit for the annual sand mining activity within the study area (see Discussion for details).

Because high flow velocities during the wet season affected safe navigation of the survey vessel, only selected mining sites (see *MS01* to *MS03* in Fig. 1a) were revisited again in October 2018. At these locations, the observed bed elevation changes between the dry and wet season indicate that the riverbed can reach a new equilibrium after only a few months. The cumulative sedimentation within the dredged pits was as high as 14 m, often accompanied by an erosion of the surrounding bathymetry (Fig. 5). Accordingly, gradients within the bathymetry were visibly smoothed by the time of the second survey (see Fig. 5j). To calculate the recovery time, i.e. the duration of the refilling process, a hydro-morphodynamic numerical model was set-up and validated based on these observations (see Supplementary Material S1). According to the model, a complete refilling within one year is likely for most mining sites, if undisturbed by continuous dredging activity. Oftentimes, the recovery time is even considerably smaller (<6 months). At the remaining locations, the refilling process is also far advanced after one year.

The results also show that local bed load transport only marginally contributes to the local refilling rates. Even the highest observed bed load transport rate of 9.61 kg/s for the wet season (Supplementary Fig. S8) would just lead to around 0.16 Mm^3^/yr being transported as bed load annually. The observed refilling and associated adjustment of the bathymetry thus must be mainly attributed to the trapping of suspended sediments and local aggregate redistribution. Due to the fine nature of the suspended sediments within the Mekong River^45–47^, it can be assumed that the bulk of the refilling material actually stems from local bed aggregates.

## Discussion

The estimated rates of suspended transport are in agreement with results from previous years^48^. Calculated bed load transport rates are similar to reported rates for the Hau River branch^39^. Bed load transport rates were indirectly calculated based on dune fields *DF01* and *DF02*, which were located downstream from most of the investigated sand mining sites. Nevertheless, it is assumed that the results for *DF01* and *DF02* are valid for the whole study area. This assumption appears more than reasonable, since bed load can be described as a function of water and sediment properties as well as hydraulic forcing^49,50^. Since hydraulic forcing and riverbed aggregates are comparable for most of the study area (see Supplementary Fig. S2), bed load transport rates must also be similar. Comparing suspended and bed load transport rates, the contribution of bed load to the total local sediment transport must be considered only minimal (<1%).

A substantial number of uncertainties are inherent to the estimated volume of annual sand mining in this study. (a) As the actual state of the undisturbed bathymetry was unknown, residual bathymetries were necessary to approximate the volume of single extraction sites. The quality of this approximation can hardly be verified. (b) The algorithm used to detect sand mining sites from the surrounding bathymetry neglected smaller sites with an area <2,500 ${m}^{2}$ (see Methods for details). Their cumulative volume is absent from the total sand mining volume. (c) As validated by a supplementary numerical model (Supplementary Material S1), mining sites can be completely refilled within only a few months. All relevant mining activities for an annual budget thus cannot be covered by one single bathymetric map only. The result is an underestimation of the actual sand mining volume. Based on the results of the numerical model, it can also be hypothesised that former mining sites should not be visible within bathymetric datasets after one full year, if unoccupied by dredgers. Taking all these points into account, the volume of 4.64 $\pm$ 0.31 Mm^3^ should be considered as a highly conservative estimate of sand mining activity within the study area for the year 2018. Despite all exposed uncertainties, it must also be highlighted that this is the first estimate of sand mining volumes for the Mekong River using high-resolution bathymetric maps of sand mining sites and the analysis of their morphological evolution. Previous estimates relied on remote sensing techniques and questionnaires^22^ or low resolution datasets^23^, which have ever since been put into question^51^.

**Figure 6:**
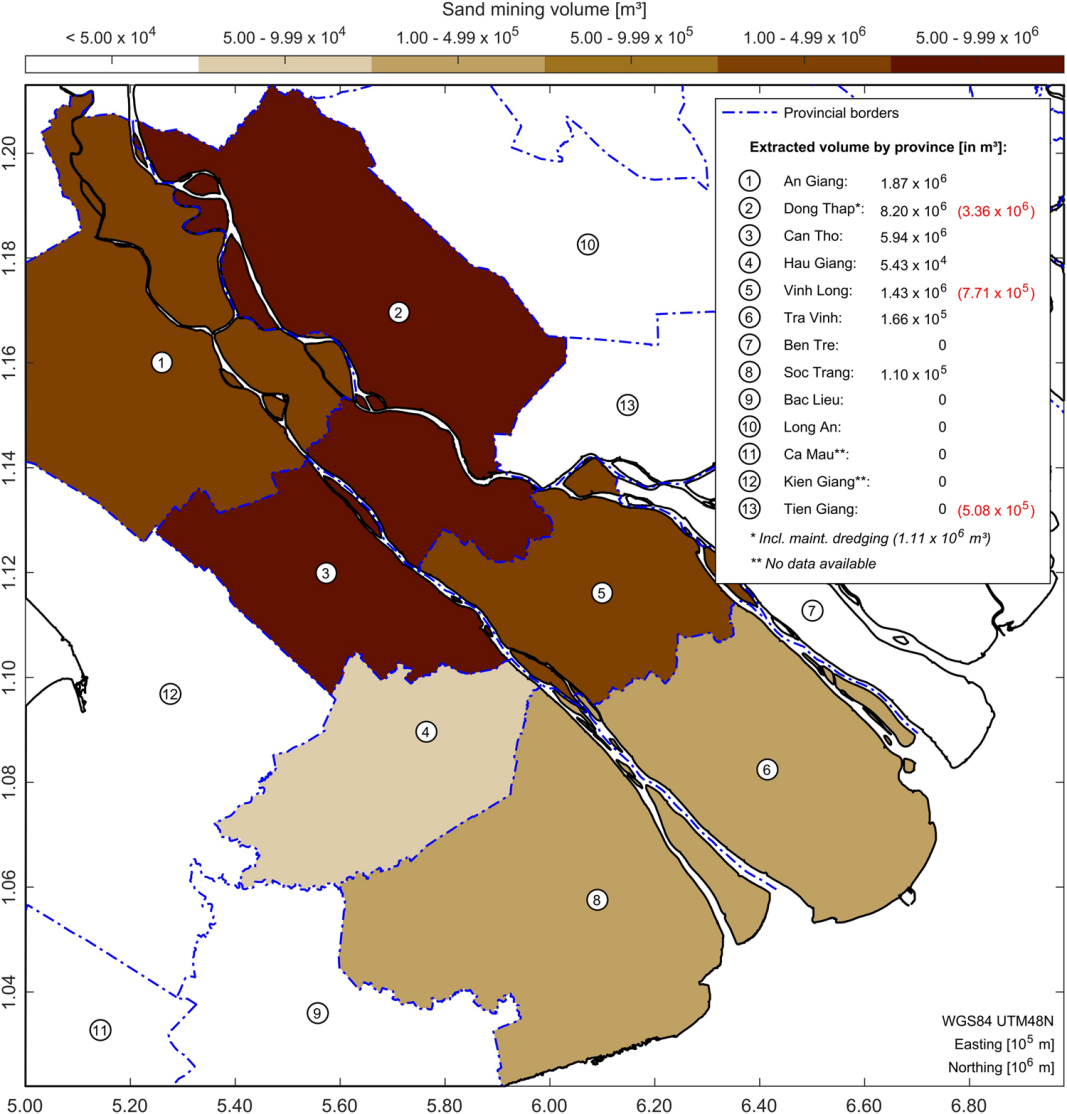
Reported statistics of extracted sand volumes within the VMD for the year 2018. Red numbers denote estimates for the provinces Dong Thap, Vinh Long and Tien Giang, based on 2018 field surveys. Provincial borders were available via the Humanitarian Data Exchange (HDX) (https://data.humdata.org/), provided by the Pacific Disaster Center (PDC). Sand mining data was collected from the responsible DONREs, the SMCD and the MONRE. Illustration was generated using Matlab 2018a (http://mathworks.com).

For the year 2018, the estimates from this study were also compared to reported statistics of sand mining activities for the whole VMD. These statistics were collected from the responsible Departments of Natural Resources and Environment (DONREs), the Southern Mineral Control Department (SMCD) as well as the Ministry of Natural Resources and Environment (MONRE) and reflect volumes, which were documented by local dredging contractors and reported to the local authorities. Accordingly, 17.77 Mm^3^ of sand were extracted within the whole VMD from the Mekong’s channels during the year 2018 (see Fig. 6). Most of the volume was extracted for the purposes of the local construction industry, while only a small amount of sand was dredged to maintain the river’s channels. According to this dataset, hot spots of dredging activity were located in the provinces Dong Thap, Can Tho, An Giang and Vinh Long. In some provinces (e.g. Ben Tre and Tien Giang), no dredging licenses were awarded and sand mining was completely prohibited for the year 2018. Calculated volumes from this study, which were assigned to the affected provinces, showed a discrepancy compared to these statistics. Some of the surveyed mining sites, which were occupied by dredgers during the 2018 dry season, were at least partly located within the Tien Giang province. The observed extraction volume for this province was around 0.51 Mm^3^. According to the statistics, dredging was completely prohibited for this province and the amount of sand mining equalled zero. This discrepancy is an indicator for informal sand mining practices within the study area, e.g. dredging without valid licenses or a violation of license regulations. It must also be highlighted that the study area only covered 20 km of the Tien River branch, while all of the Mekong’s channels within the VMD have a cumulative length of several hundreds of kilometres. Based on the observations, the actual numbers for the year 2018 are likely to be significantly higher than the reported 17.77 Mm^3^ if one assumes a multitude of similar hot spots across the whole delta, as previously reported^22,23^. Compared to existing estimates of sand mining activities for the entire VMD, which were of the order of 7.7 to 20 Mm^3^/yr^22,23^, this would also mean an increase in dredging activity in recent years, or a substantial underestimation of the extraction rates in previous studies.

In addition, contrasting the observed extraction volumes to the local sediment transport rates, the non-sustainable practices of the sand mining activities within the Tien River study area are clearly pointed-out. Even when applying the highest bed load transport rate observed during the wet season, bed load transport could only compensate for around 3% of the extracted aggregates. According to ref. 39, the annual suspended sand fluxes within the Tien River are of the order of 3.8 Mt/yr, representing around 44% of the observed sand extraction. Only a fraction of this suspended load effectively contributes to the refilling process, depending on the local sediment and flow characteristics as well as the geometry of dredging pits. The total sand flux (bed load + suspended load) thus cannot compensate for the extracted amount of sand, leading to a substantial annual deficit, which should be even more pronounced if up-scaled for the whole Tien River branch.

## Conclusion and Outlook

Generally, extraction pits due to sand mining act as a sediment sink for river aggregates. If sediment transport rates are too small to compensate for sediment losses due to mining activities, the relation between sediment supply and transport capacity becomes distorted. The consequent sediment starvation within the water acts as a trigger for erosion processes. As shown here, the anthropogenic induced imbalance between sediment supply and sand mining activities can be substantial within the VMD. Resulting incision of river channels and bank erosion, which have previously already been reported for the Mekong River^23,29,30^, are strikingly exposed for a 20 km stretch of the Tien River, which was investigated by this study. Even though it seems impossible to quantify the detailed impact of sand mining on the local morphological processes, it is indisputable that sand mining is an important cause for the changing environment within the VMD and a trigger for erosional processes.

On a large scale, the reduction of downstream sediment supply, resulting from the cumulative effects of sand mining, dam construction and changes in the local cyclone activity^28^, put the delta’s future stability into question. The manifold consequences, which include bank erosion^29,30^, recessing coastlines^31–33^, increasing salinity intrusion^27^ and a loss of mangrove forests^34^, will likely only accelerate if current sand mining practices are maintained in the future. To ensure sustainable development within the delta region, the overarching objective must be the implementation and enforcement of regulations that mitigate the net loss of sediments due to sand mining. In particular, local sand mining practices should be monitored and controlled in more detail to prevent informal activities. Reliable data is of particular importance to understand the relations between the sand mining and the triggered processes. Accordingly, continuous measurements and numerical models should also be used to reduce existing uncertainties and provide more insight into the links between the underlying hydro- and morphological processes. These models could also project the effects of possible future scenarios for the local extraction of bed aggregates.

## Methods

### Bathymetry mapping

Bathymetric surveys were conducted with a Reson Seabat 7125 AUV MBES coupled to a motion sensor, a sound velocity probe (SVP) and a global navigation satellite system (GNSS) positioning unit, which were installed on board a local survey vessel. Data from the MBES and external sensors was recorded and merged within the PDS2000 software (version 3.4.0.1). The vertical accuracy of the system, resulting from all sensors, was of the order of <0.1 m. Offsets for mounting angles for roll, pitch and heading were calibrated based on the BeamworX software AutoPatch (version 2018.2). Raw datasets were processed and gridded using the BeamworX software AutoClean (version 2018.2). This resulted in digital elevation models (DEMs) with a resolution of 1 $\times$ 1 m. Heights of DEMs were referenced to the Earth Gravitational Model 2008 (EGM2008).

### Calculation of sand mining volumes and refilling rates

To separate mining sites from the surrounding bathymetry, the optimal robust separator (ORS) algorithm^52^ was applied. Before filtering of the original surveyed bathymetry, empty depth cells landward of the land-water interface were padded with zeros. The bank line from January 29, 2017, which was extracted from satellite images, was therefore used to define the land-water interface. The height for remaining empty depth cells was linearly interpolated. Afterwards, the original bathymetry was filtered by means of a median filter using the Generic Mapping Tools (GMT) (version 6.0.0) function grdfilter. Different filter widths ${w}_{i}$ were used, incrementally increasing the diameter by 100 m. For each diameter, the resulting regional bathymetry ${f}_{i}(x,y)$ was subtracted from the observational dataset $s(x,y)$ to calculate a residual signal ${d}_{i}(x,y)$. A depth contour of $-1$ m was then chosen to identify polygons reflecting the sand mining sites within these residual datasets. Resulting polygons with an area <2,500 ${m}^{2}$ were neglected, since the associated mining sites could not always be clearly identified. Additional polygons were deleted during visual inspection. The ORS was then used to calculate the residual height $\bar{{h}_{i}}$ as follows:

1 $$\bar{{h}_{i}}=\frac{{V}_{i}}{{A}_{i}}$$

where ${A}_{i}$ is the area of the residual bathymetry enclosed by the $-1$ m depth contour and ${V}_{i}$ is the volume under the residual for this area. For each mining site, suitable regional bathymetries were selected for the separation of the mining site from the surrounding bathymetry. In order to achieve this, the regional bathymetry leading to the maximum residual height as well as the ones associated with the next smaller and larger diameters were considered suitable candidates. In some cases, the increase in filter diameter led to a residual bathymetry, where single mining sites conflicted with adjacent bathymetric features, e.g. bedforms or scour holes. Since the spatial extent of mining sites could not be determined in such cases, the concerned regional bathymetries were neglected as possible candidates. Using the approach by ref. 53, the separation of a mining site from the surrounding regional bathymetry was afterwards achieved by following procedure: First, the final regional bathymetry for a location ${f}_{m}(x,y)$ was obtained by calculating the median of all selected candidates ${f}_{c}(x,y)$ as follows:

2 $${f}_{m}(x,y)=median| {f}_{c}(x,y)| $$

To assess the sensitivity of the results to different filter diameters, the median absolute deviation (MAD)^54^ ${\sigma }^{* }(x,y)$ was calculated as:

3 $${\sigma }^{* }(x,y)=1.482\times median| {f}_{c}(x,y)-{f}_{m}(x,y)| $$

The final residual bathymetry ${d}_{m}(x,y)$ was obtained by subtracting the final regional bathymetry from the observational data and using the MAD to provide uncertainty bounds:

4 $${d}_{m}(x,y)=s(x,y)-[{f}_{m}(x,y)\pm {\sigma }^{* }(x,y)]$$

**Figure 7:**
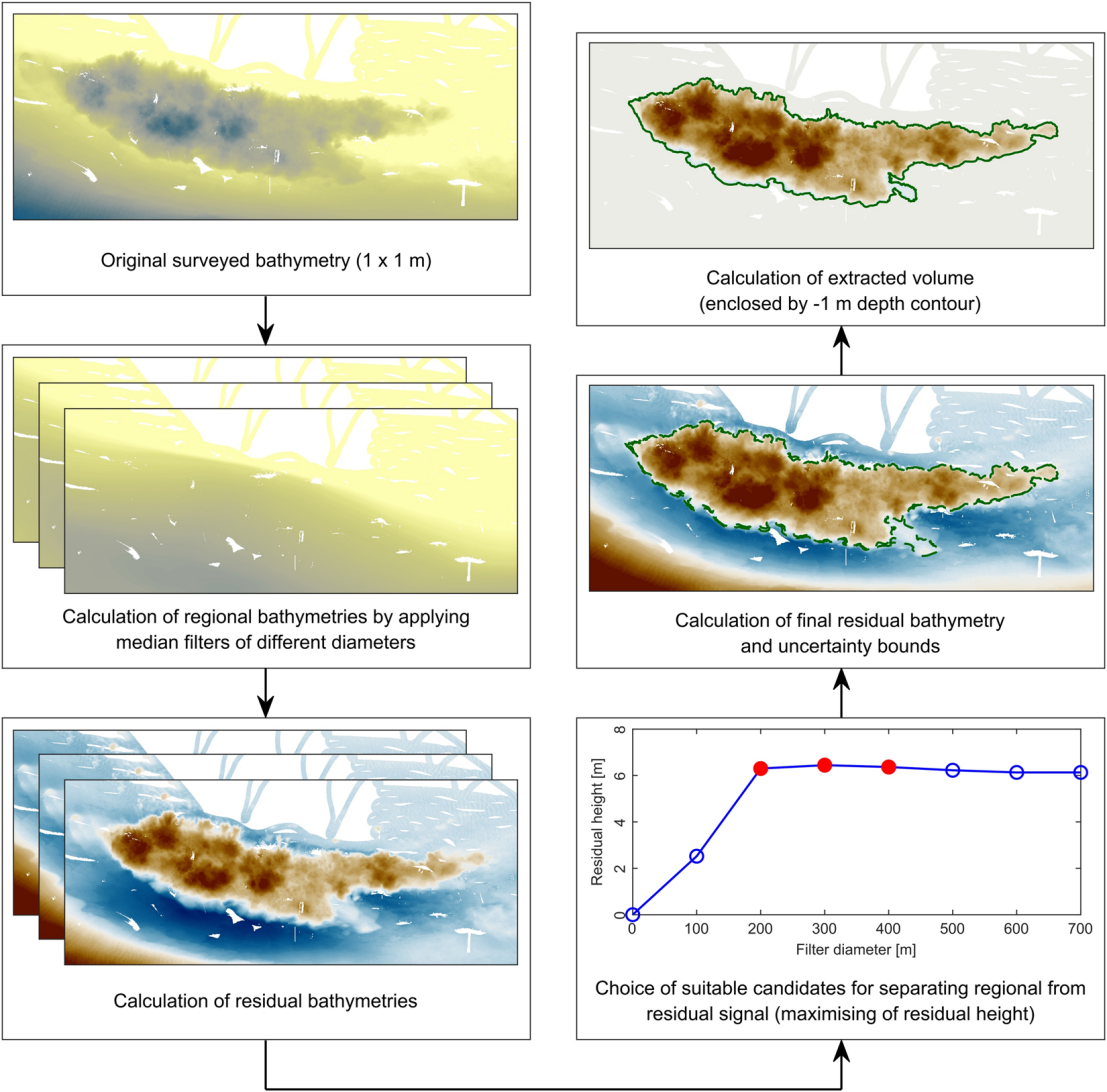
Procedure for calculating extracted sand volume for all mining sites within the study area. Illustration was generated using Matlab 2018a (http://mathworks.com).

The extraction volume for each mining site was then estimated based on the volume under ${d}_{m}$ enclosed by the $-1$ m depth contour of this final residual bathymetry. The detailed procedure is also shown in Fig. 7. The recovery time, i.e. the duration of the refilling process of local sand mining sites, was calculated by means of a supplementary numerical model using the software Delft3D^55^. A detailed description of the set-up, validation and the results of this model can be found in the Supplementary Material S1.

### Calculation of bed load transport rates

Non-intrusive approaches for the estimation of bed load transport rates usually combine measured dune geometries and migration rates with sediment properties^44,56,57^. While most of these approaches rely on a single longitudinal cross-section of a dune field^56,57^, the bed load translation method^44^, which was applied within this study, uses high-resolution bathymetric datasets from subsequent MBES surveys. Patterns of erosion and accretion are therefore used to perform three-dimensional calculations, deriving dune migration rates and changes in bed elevation. Height offsets, arising from the vertical accuracy of the MBES system, which were initially observed between datasets, were removed. Afterwards, bed level changes between subsequent datasets were calculated as:

5 $$\Delta z=Z({t}_{2})-Z({t}_{1})$$

where $Z({t}_{1})$ and $Z({t}_{2})$ are the bed elevation of subsequent datasets and $\Delta z$ is the elevation change per bathymetric cell. Concentrating on the lee face deposition and the associated mean height change per cell $\langle \Delta z\rangle$, the migration distance of dunes is defined by:

6 $$\langle \Delta X\rangle =\frac{\sum area[Z({t}_{2}) < Z({t}_{1})]}{{N}_{bedforms}\times {W}_{dune}}$$

where ${N}_{bedforms}$ is the number of bedforms within a defined polygon and ${W}_{dune}$ is the effective polygon width (i.e. the length of the dune crest line). The channel mass flux can then be calculated as:

7 $${Q}_{s}=\langle {\varepsilon }_{bed}\left(\frac{\langle \Delta X\rangle \times \langle \Delta z\rangle }{\Delta t}\right)\rangle \times \rho \times \omega $$

where ${\varepsilon }_{bed}$ is the volume concentration of sediment in the bed, $\Delta t$ is the time between subsequent surveys, $\rho$ is the sediment density and $\omega$ is the width of the dune field. Within this study, a porosity of 30% (i.e. ${\varepsilon }_{bed}$ = 0.7) and a sediment density of 2,650 kg/${m}^{3}$ was applied for all conversions between sediment volume and sediment mass. Due to the three-dimensional nature of bedforms within investigated dune fields, their number and respective widths could not be easily determined. Thus, longitudinal sections were defined along a dune field for every 5 m of width. The bedform tracking tool^58^ was applied to identify the crests of primary dunes along each section. Identified crests were connected upon visual inspection within QGIS (version 2.18.11). The crest lines were then used to calculate the total length of all dunes within a field. Finally, an underestimation of the transport rates, which is inherent to the bedform translation method, was corrected based on the ratio of observed dune translation to overall dune length (see ref. 44 for details).

### Processing of satellite images

Locations of riverbank erosion within the study area were identified based on exemplary historical satellite imagery with a cloud cover of less than 10%. Therefore, the modified normalised difference water index (MNDWI)^59^ was applied within QGIS using openly available Landsat-7 and 8 images. First, the Semi-Automatic Classification Plugin^60^ was used to calculate top-of-atmosphere (TOA) reflectance from raw digital numbers. Afterwards, the green and short-wave infrared bands were used to calculate the $MNDWI$ as:

8 $$MNDWI=\frac{{\rho }_{Green}-{\rho }_{SWIR1}}{{\rho }_{Green}+{\rho }_{SWIR1}}$$

where ${\rho }_{Green}$ and ${\rho }_{SWIR1}$ are the TOA reflectance of the green and short-wave infrared bands. A threshold of 0.4 was used to delineate water from land mass. Some features (e.g. clouds) that did not reflect the actual bank lines were removed upon visual inspection. In order to identify areas of bank erosion and accretion based on historical satellite images, a centre line was defined using the river’s present-day geometry. Every 100 m along the centre line, orthogonal lines were drawn. The intersection points of these orthogonals with the shapes of the historical riverbanks were used to assess riverbank migration. These changes in riverbank location were plotted against their respective position along the RKM. Afterwards, high-resolution satellite images from Google Earth were used to check whether areas of land accretion corresponded to locations with aquaculture infrastructure. The identified areas were highlighted separately.

## Supplementary information

Supplementary information

## Data Availability

Relevant bathymetrical datasets will be made available on PANGAEA (https://www.pangaea.de/). Other selected datasets are available via the Mekong Knowledge Hub (https://catchmekong.eoc.dlr.de/Elvis/) or can be acquired from the corresponding author on reasonable request.
