# Supplementary material for: Sand mining in the Mekong Delta revisited - current scales of local sediment deficits
Source: Sci Rep. 2019 Nov 28;9:17823. doi: 10.1038/s41598-019-53804-z (PMC6882805; doi:10.1038/s41598-019-53804-z)
Supplement: Supplementary file 1 — Supplementary information [file 41598_2019_53804_MOESM1_ESM.pdf]

Supplementary information

Sand mining in the Mekong Delta revisited - current scales of local sediment deficits

Christian Jordan[1,*], Jan Tiede[1], Oliver Lojek[1], Jan Visscher[1], Heiko Apel[2], Hong Quan Nguyen[3], Chau Nguyen Xuan Quang[4], Torsten Schlurmann[1]

[1]Ludwig-Franzius-Institute for hydraulic, estuarine and coastal engineering, Leibniz University Hannover, Hannover, 30167, Germany
[2]GFZ - German Research Center for Geoscience, Section Hydrology, Potsdam, 14473, Germany
[3]Center of Water Management and Climate Change, Institute for Environment and Resources, Vietnam National University, Ho Chi Minh City, 700000, Vietnam
[4]Department of Hydrology and Water Resources, Institute for Environment and Resources, Vietnam National University, Ho Chi Minh City, 700000, Vietnam
[*]jordan@lufi.uni-hannover.de

The supplementary material contains following items:

S1. Calculation of recovery time

S2. Supplementary figures

S3. Supplementary tables

S1. Calculation of recovery time

Model set-up

In order to validate the observed refilling process, a hydro-morphodynamic model of the study area was set-up using Delft3D[1], covering the area between river kilometre markers (RKMs) 7 to 16, where most of the sand mining sites were located. Delft3D solves the Reynolds-averaged Navier-Stokes equations on a staggered grid, using a finite-difference scheme. A detailed description of the used formulations and implementations can be found in refs 1, 2.

The model domain of the hydro-morphodynamic model has a horizontal grid resolution of around 10 x 20 m (Supplementary Fig. S1). Bathymetry data was interpolated onto the numerical grid, based on measurements merged with TandDEM-X[3] topography data. Discharges and water levels, which were available for the location My Thuan (Vietnam) for the whole year 2018, were used to generate Riemann boundaries as hydrodynamic forcing along two open boundaries. The Earth Gravitational Model 2008 (EGM2008) height reference system was used as vertical datum. Sediment transport was modelled with the van Rijn TR2004 equation[4]. Due to the lack of measured sediment concentrations for the duration of the simulation, equilibrium concentrations were used to reconstruct sediment input along the open boundaries. The bed sediment itself was simplified by taking a representative single sand fraction with a median diameter of 200 µm. This is reasonable, since an analysis of sediment samples revealed that fine to medium sands dominate along the riverbed of the study area (see Supplementary Fig. S2). The contribution of cohesive sediment fractions with even smaller grain-sizes was neglected due to the prevailing hydrodynamic conditions. This simplification is supported by the fact that even low approach velocities of 0.2 m/s in combination with commonly used sinking velocities of 0.05 to 0.08 mm/s[5–7] would only lead to negligible trapping efficiencies, according to ref. 8. To calibrate the model, the bed roughness was used as a tuning parameter. A range of different Manning (n) coefficients was tested based of refs 5, 6, 9, resulting in a uniform coefficient of $0.024\,\mathrm{s/m^{1/3}}$.

Model validation

To validate the hydrodynamics within the model, simulations were initially run in three-dimensional configuration with 20 vertical layers. For this configuration, the model performance was compared with selected acoustic Doppler current profiler (ADCP) measurements, which were conducted on May 18, 2018 along transect I-J (see location in Supplementary Fig. S1). To quantify the performance, the relative error vector (REV)[10] was used to evaluate measured and simulated velocity vectors. The *REV* can be calculated as:

$$REV = \frac{\langle \sqrt{(u_{\mathrm{meas}} - u_{\mathrm{calc}})^2 + (v_{\mathrm{meas}} - v_{\mathrm{calc}})^2} \rangle}{\langle \sqrt{(u_{\mathrm{meas}} + v_{\mathrm{meas}})^2} \rangle} \tag{1}$$

where u_{meas} and v_{meas} are the measured flow velocities in horizontal x- and y-direction, while u_{calc} and v_{calc} are the corresponding simulated flow velocities. Angle brackets indicate the spatial averaging of mentioned parameters. According to the *REV*, values < 0.2 indicate excellent model performance (see Supplementary Table S1), while values < 0.4 reflect good model performance. Even though only a small number of observations were available to validate the reproduction of hydrodynamic processes within the model, the results show satisfying results (Supplementary Fig. S3). Since two- and three-dimensional simulations showed negligible differences in the detected morphological changes, the model was run in 2D depth averaged mode after the hydrodynamic validation in order to save computational time. To validate the model performance for these simulations, the Brier skill score (BSS)[11] was used, which evaluates modelled and measured anomalies. The *BSS* is defined by:

$$BSS = 1 - \frac{\langle (Y - X)^2 \rangle}{\langle (B - X)^2 \rangle} \tag{2}$$

where B is the initial bathymetry at the start of a simulation, Y is the simulated bathymetry at the end of a simulation and X is the corresponding measured bathymetry at the same time. The *BSS* was calculated for mining sites *MS01* to *MS03* (corresponding to locations 2, 5 and 7 in Supplementary Fig. S1), which were surveyed in detail during both the dry and wet season 2018. At these locations, the simulated refilling process was compared to observational data for the period from May 10 to October 10, 2018. Here, a $BSS > 0.5$ indicates excellent model performance (see Supplementary Table S1), while values > 0.2 are equivalent to good model performance. Thus, the model shows favourable results simulating the refilling process at locations *MS01* and *MS02* (Supplementary Fig. S4). According to a $BSS < 0$, the model performance for *MS03* is bad with reference to the validation data. The unsatisfying performance at this location results from not considering the dredging activities within the numerical model, since detailed information about the operation of dredgers between May to October 2018 was lacking. Thus, the model results reflect the undisturbed refilling process, while the validation data includes dredging

activities. The measurements indicate that continuous dredging activity was disturbing the refilling process at mining site *MS03*, leading to the discrepancies between model results and observations. Accordingly, the overall performance of the model regarding hydro-morphodynamic processes was considered good.

Results

After the validation process, the model was used to determine the recovery time, i.e. the duration of the refilling process for all mining sites located within the model domain. Therefore, the model was run for one and a half years, starting on May 18, 2018. Since the flow velocities and water levels were not available beyond December 2018, the hydrodynamic conditions for the year 2018 were extended periodically. The morphological changes within the model domain were evaluated in detail. After the completion of the refilling process, sedimentation reverted to erosion at some locations while the bed elevation remained nearly constant at other locations, indicating a new equilibrium. For each mining site, the maximum cumulative sedimentation was determined for the lowest point beneath the surrounding bathymetry. The end of the refilling process was defined as the time t_{i}, when 95 % of this maximum was exceeded. After this limit was reached, the height changes per day stayed low for the remainder of the simulation. According to this definition, the refilling process had ended at almost every mining site within the model domain after one full year (see Supplementary Table S2). Generally, the recovery time was already reached after less than 6 months, i.e. after the end of the first wet season. Only at locations 3 and 7, the refilling process was far advanced but not yet completed after one full year. Here, it was evident that the recovery time would be reached by the end of the second wet season. According to this results, it is more than plausible that the observed hydrodynamics for the year 2018 lead to a recovery time of less than one year, if the refilling process is undisturbed by continuous dredging activity. Only on rare occasion, the combination of local flow and sediment characteristics as well as the geometry of single sand mining sites will lead to a longer recovery time. Even for those cases, remnants of dredging activity should only be hardly identifiable after one year. However, it must be noted that the discharge during the wet season 2018 was higher compared to previous years (see Supplementary Fig. S5). A complete refilling thus was more likely to occur than for an average year.

S2. Supplementary figures

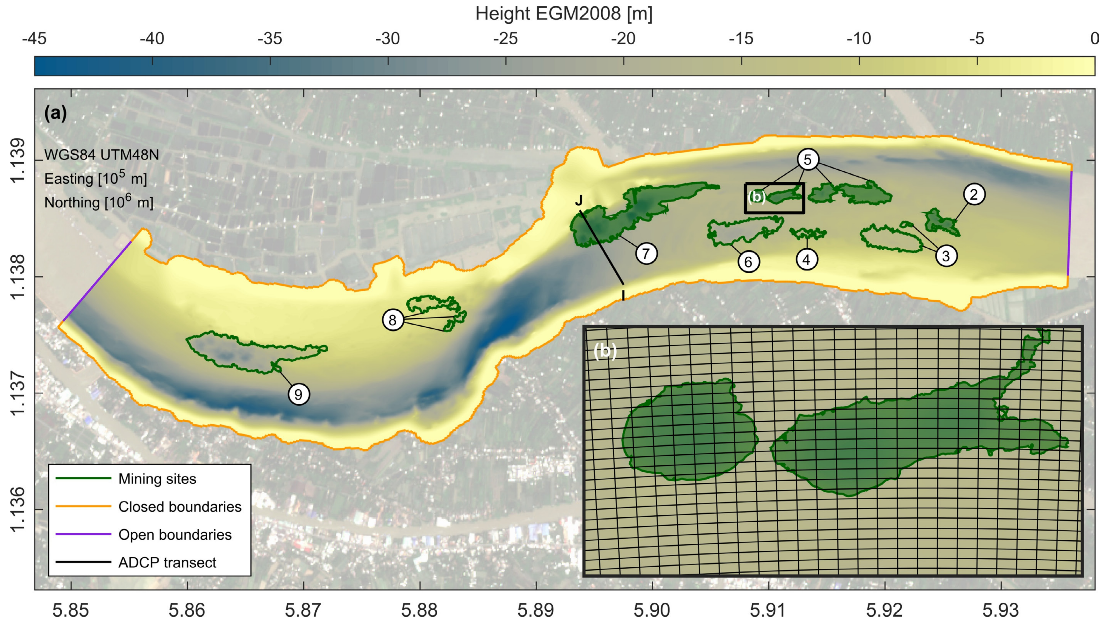

Supplementary Figure S1. Domain of the supplementary numerical model. (a): Overview of model domain with locations of mining sites, boundaries and ADCP transect. Sentinel-2 data from October 28, 2018, was used as background image. (b): Detailed view of the numerical grid at location 5. Mining sites *MS01* to *MS03* correspond to locations 2, 5 and 7, respectively. Sentinel-2 (ESA) image courtesy of the U.S. Geological Survey (USGS), downloaded from the USGS Earth Resources Observation and Science (EROS) Center (https://earthexplorer.usgs.gov/). Copernicus Sentinel data 2018, processed by ESA. Illustrations (a) and (b) were generated using Matlab 2018a (http://mathworks.com).

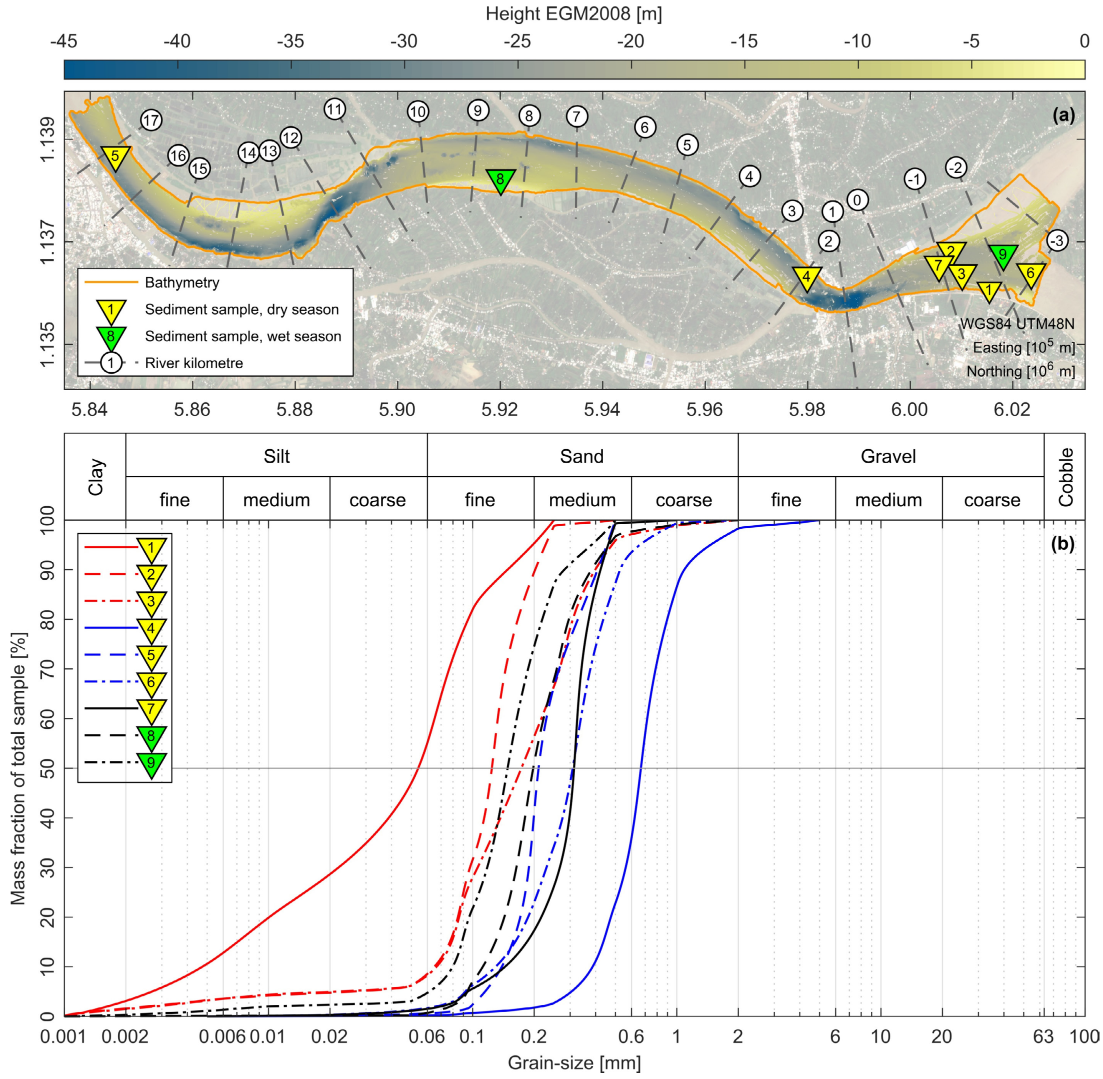

Supplementary Figure S2. Locations and grain-size distributions of bed sediment samples. (a): Map with locations of bed sediment samples taken during the dry and wet season 2018. Bed sediment samples during the wet season were directly taken from dredgers operating at locations 8 and 9. Sentinel-2 data from October 28, 2018, was used as background image. (b): Grain-size distributions of bed sediment samples. Bed samples were analysed according to the ASTM International (ASTM) D422-63 standard. Sentinel-2 (ESA) image courtesy of the USGS, downloaded from the USGS EROS Center (https://earthexplorer.usgs.gov/). Copernicus Sentinel data 2018, processed by ESA. Illustrations (a) and (b) were generated using Matlab 2018a (http://mathworks.com).

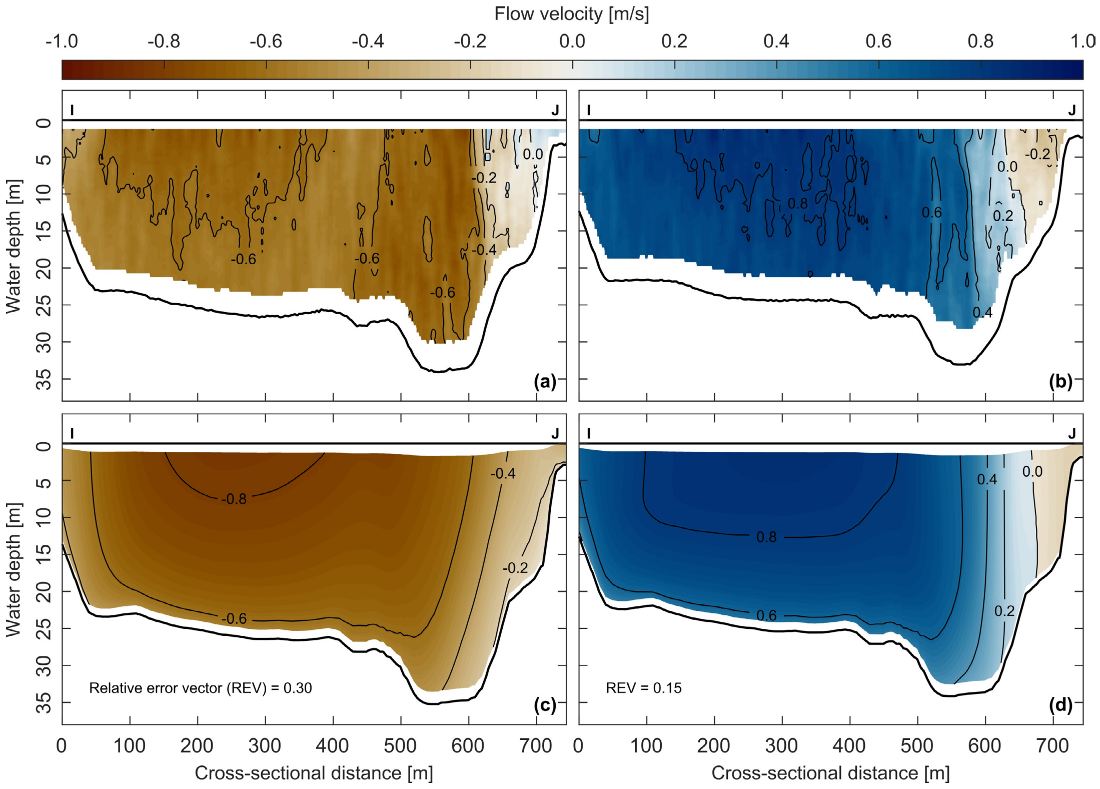

Supplementary Figure S3. Measured and simulated flow velocities along ADCP profile I-J (see location in Supplementary Fig. S1) for the dry and wet season 2018 and corresponding $REVs$. (a): Measured flow velocities around flood for May 18, 2018, 00:20 AM (UTC). (b): Measured flow velocities around ebb for May 18, 2018, 04:51 AM (UTC). (c): Simulated flow velocities around flood for May 18, 2018, 00:20 AM (UTC). (d): Simulated flow velocities around ebb for May 18, 2018, 04:51 AM (UTC). Negative values indicate velocities directed in upstream direction. Illustrations (a) to (d) were generated using Matlab 2018a (http://mathworks.com).

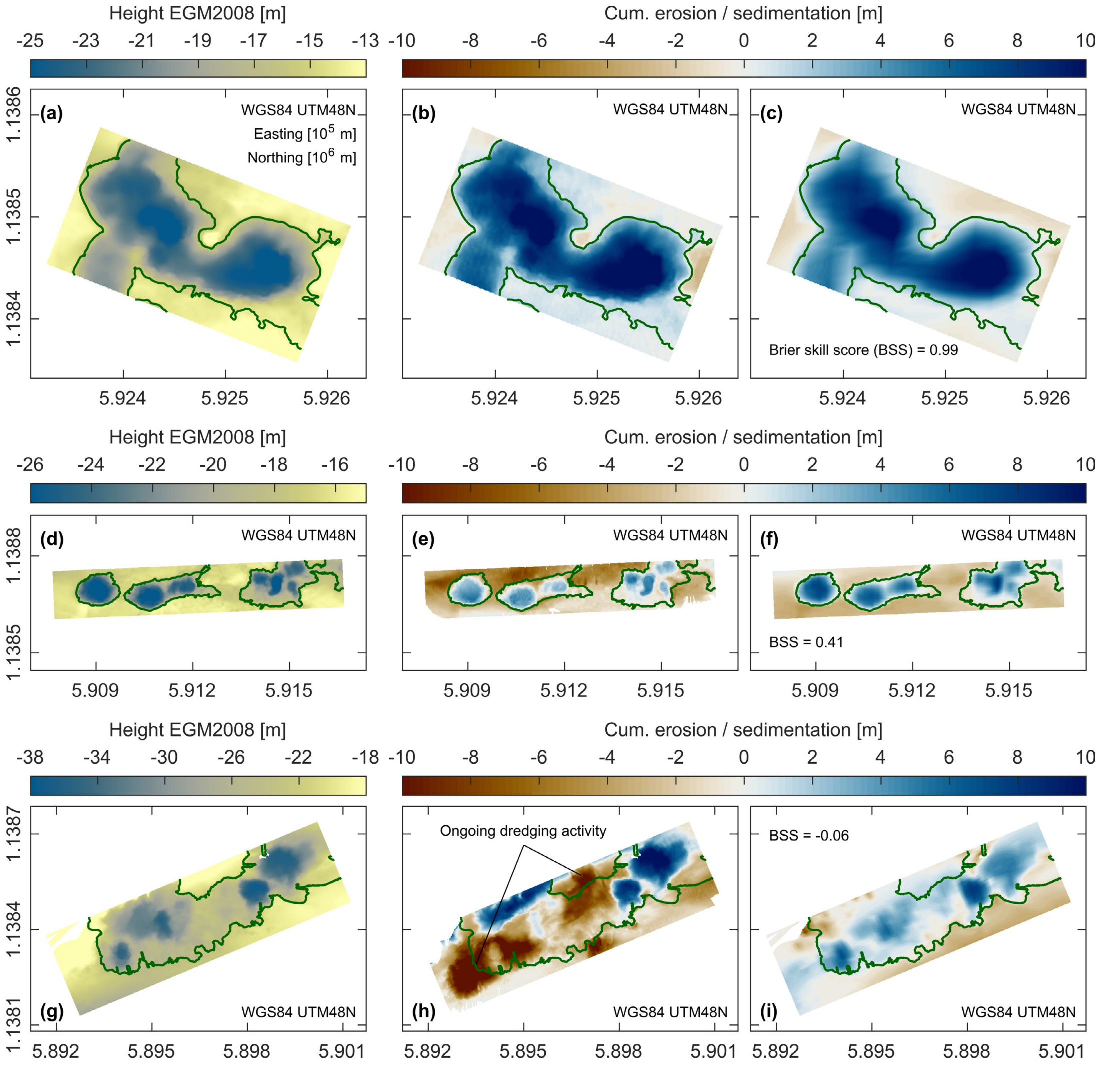

Supplementary Figure S4. Measured and simulated morphological evolution at mining sites *MS01* to *MS03* from May to October 2018 and corresponding *BSSs*. (a): Measured bathymetry at mining site *MS01* in early May 2018. (b): Measured morphological evolution at mining site *MS01* from May to October 2018. (c): Simulated morphological evolution at mining site *MS01* from May to October 2018. (d): Measured bathymetry at mining site *MS02* in early May 2018. (e): Measured morphological evolution at mining site *MS02* from May to October 2018. (f): Simulated morphological evolution at mining site *MS02* from May to October 2018. (g): Measured bathymetry at mining site *MS03* in early May 2018. (h): Measured morphological evolution at mining site *MS03* from May to October 2018. (i): Simulated morphological evolution at mining site *MS03* from May to October 2018. Note the different ranges of colourbars. Illustrations (a) to (i) were generated using Matlab 2018a (http://mathworks.com).

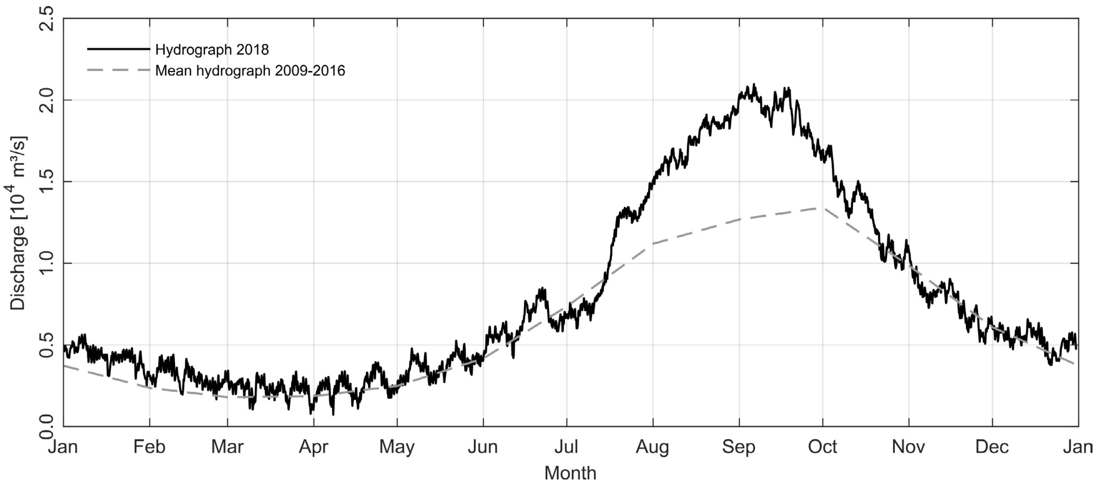

Supplementary Figure S5. Discharge hydrograph for the station My Thuan (Vietnam). Average discharge (grey dashed line) at My Thuan for the years 2009 to 2016[12]. Data for the year 2018 (black solid line) is based on a tidal-average of instantaneous discharges. Raw discharge data was provided by the Southern Regional Hydro-Meteorological Centre (SRHMC). The illustration was generated using Matlab 2018a (http://mathworks.com).

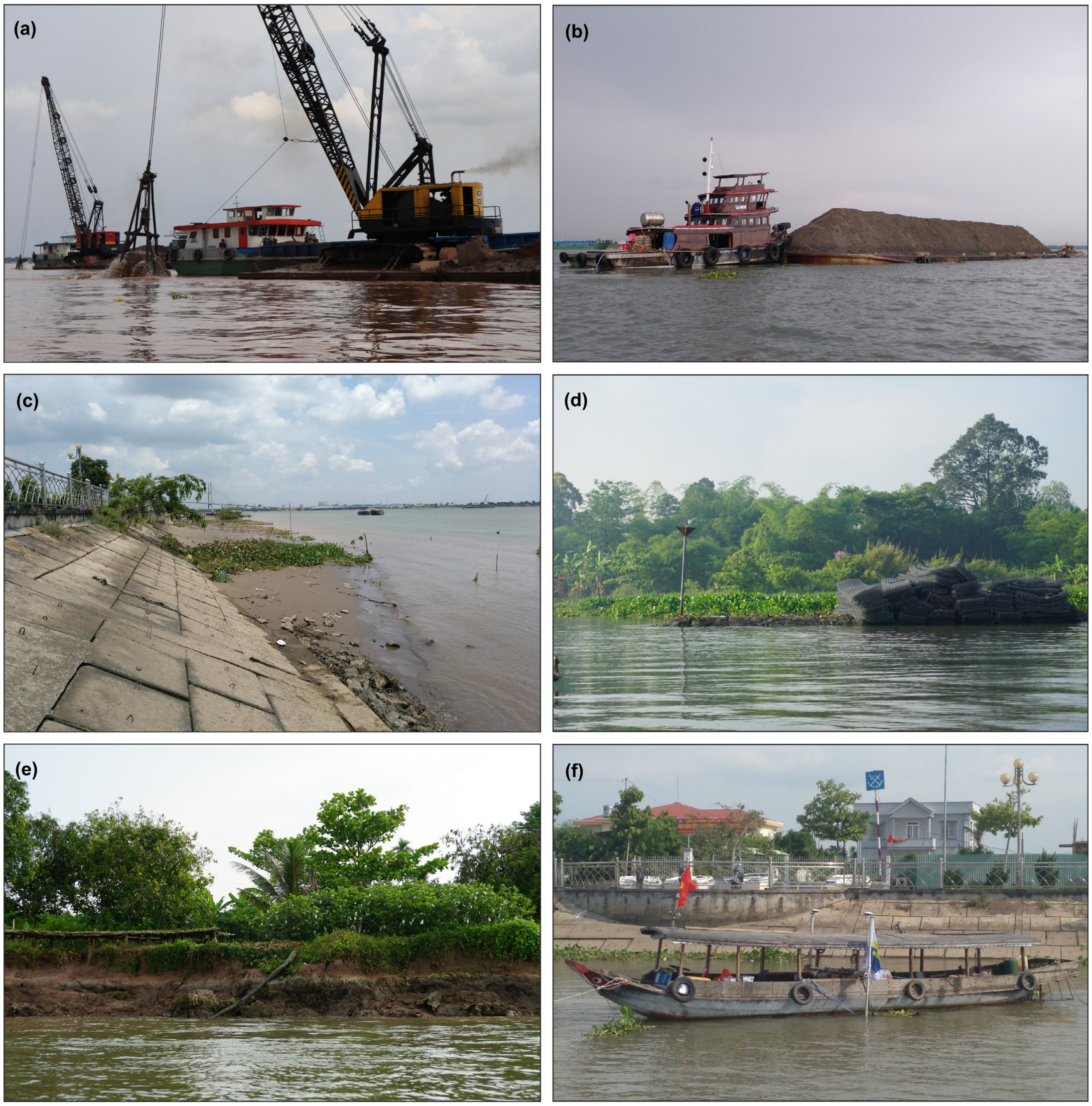

Supplementary Figure S6. Typical images of the study area taken during the dry and wet season 2018. (a): Sand-mining activities near the city of Sa Dec. (b): Barge loaded with extracted sand. (c): Revetments near the city of Vinh Long. (d): Construction of groynes to protect riverbanks from continuous erosion. (e): Continuous bank erosion near the city of Sa Dec. (f): Survey-vessel equipped with multibeam echo sounder (MBES).

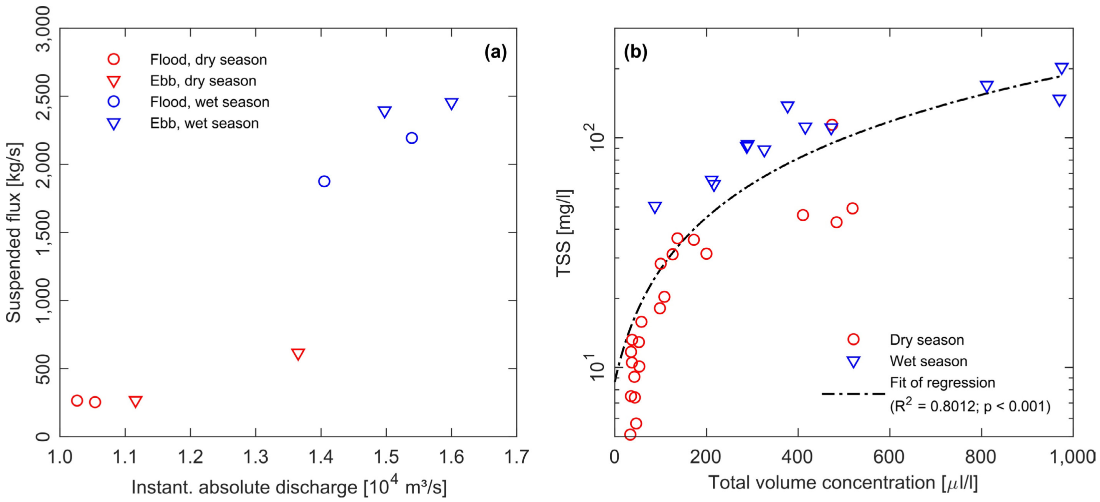

Supplementary Figure S7. Suspended load transport rates for transect A-B (see Fig. 1a, c). (a): Suspended load transport rates measured during ebb and flood for the dry and wet season 2018. (b): Relationship between volumetric concentrations and total suspended solids (TSS). Volumetric concentrations were measured via a laser in-situ scattering and transmissiometry (LISST) probe, while TSS was calculated from water samples. TSS was analysed according to the Standard Methods for the Examination of Waste and Wastewater (SMEWW) 2540 test. Note that the y-axis in panel (b) is in log scale. Illustrations (a) and (b) were generated using Matlab 2018a (http://mathworks.com).

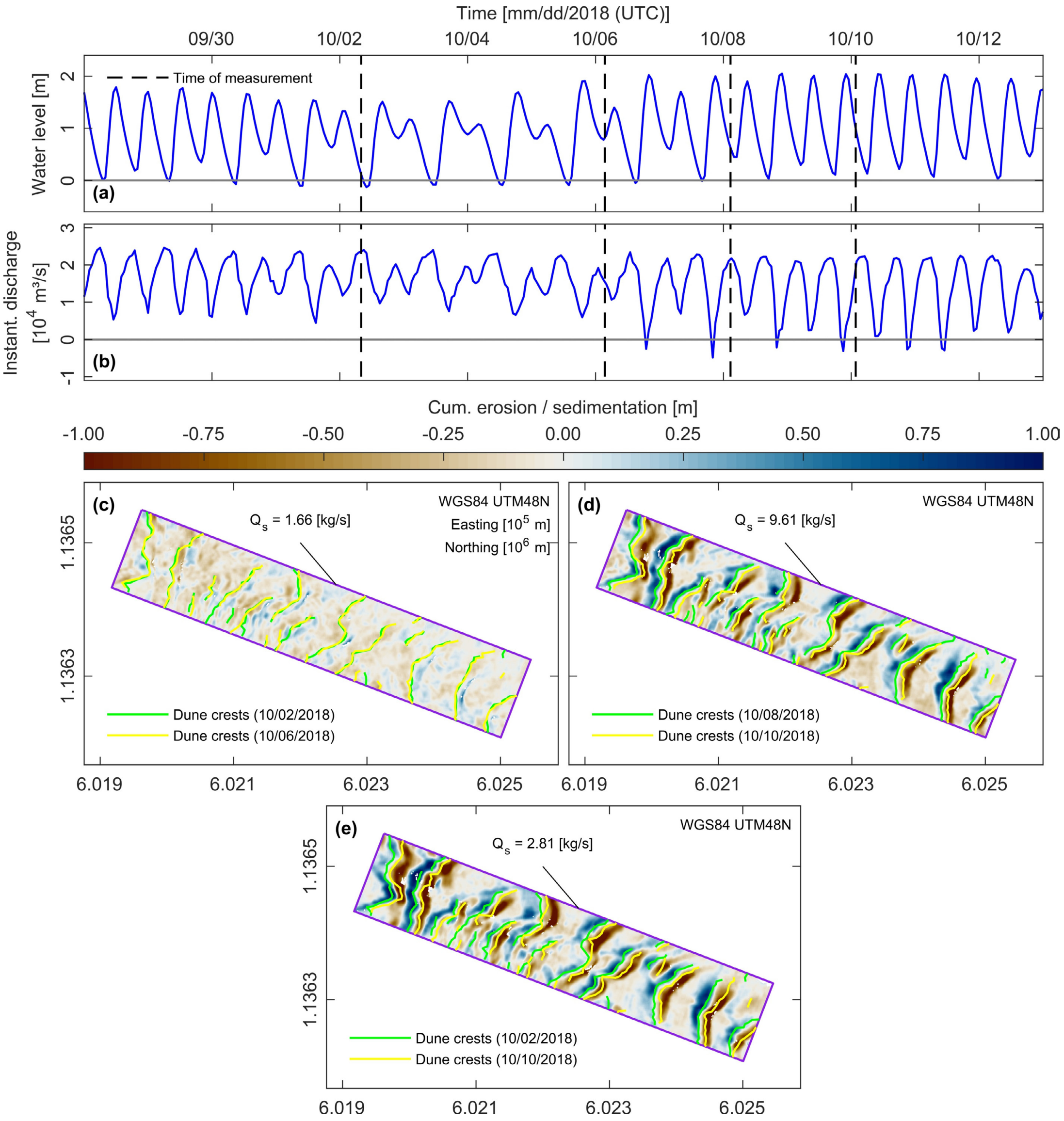

Supplementary Figure S8. Bed load transport rates based on different bathymetric datasets for dune field *DF01* and associated hydrodynamic conditions. (a): Water level from September 28 to October 12, 2018. (b): Instantaneous discharge from September 28 to October 12, 2018. (c): Bed load transport rates based on bathymetric surveys for October 2 and 6, 2018. (d): Bed load transport rates based on bathymetric surveys for October 8 and 10, 2018. (e): Bed load transport rates based on bathymetric surveys for October 2 and 10, 2018. Raw water level and discharge data was provided by the SRHMC. Illustrations (a) to (e) were generated using Matlab 2018a (http://mathworks.com).

S3. Supplementary tables

Qualification	REV	BSS
Excellent	< 0.2	1.0-0.5
Good	0.2-0.4	0.5-0.2
Reasonable	0.4-0.7	0.2-0.1
Poor	0.7-1.0	0.1-0.0
Bad	> 1.0	< 0.0

Supplementary Table S1. Error classification for the *REV*[10] and *BSS*[11].

Location	Recovery time [days]
2	131
3	>365
4	106
5	122
6	94
7	>365
8	129
9	282

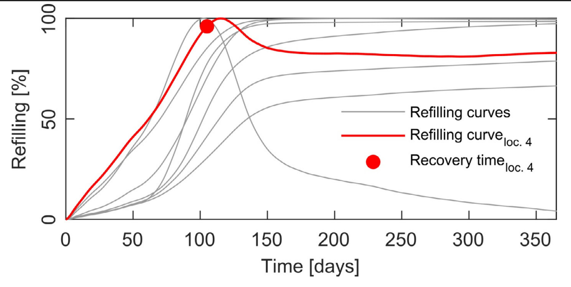

Supplementary Table S2. Calculated recovery time for all mining sites located within the model domain (see positions in Supplementary Fig. S1). The illustration shows the observed refilling curves for all mining sites. The exemplary red line shows the refilling process for location 4, with the red dot indicating the calculated recovery time. The illustration was generated using Matlab 2018a (http://mathworks.com).

Discharge parameter [$10^4 m^3/s$]	Month 2018											
	**Jan.**	**Feb.**	**Mar.**	**Apr.**	**May**	**Jun.**	**Jul.**	**Aug.**	**Sep.**	**Oct.**	**Nov.**	**Dec.**
Max. $Q_{Instant.}$	2.02	1.87	1.76	1.65	1.73	1.90	2.22	2.56	2.84	2.40	1.99	1.90
Min. $Q_{Instant.}$	-1.39	-1.41	-1.50	-1.48	-1.33	-1.11	-1.09	-0.38	0.46	-0.84	-1.05	-1.35

Supplementary Table S3. Evolution of instantaneous and riverine discharge at the station My Thuan (Vietnam) over the seasonal cycle 2018. Raw discharge data was provided by the SRHMC.

Water level parameter [m]	Month 2018											
	**Jan.**	**Feb.**	**Mar.**	**Apr.**	**May**	**Jun.**	**Jul.**	**Aug.**	**Sep.**	**Oct.**	**Nov.**	**Dec.**
Mean high-water	1.22	1.18	1.11	1.05	0.99	0.85	0.98	1.19	1.42	1.56	1.37	1.27
Mean low-water	-0.34	-0.41	-0.58	-0.66	-0.73	-0.76	-0.60	-0.19	0.07	0.11	-0.14	-0.24
Mean tidal range	1.55	1.58	1.68	1.72	1.72	1.63	1.58	1.38	1.34	1.44	1.52	1.51
Mean water level	0.45	0.39	0.28	0.22	0.13	0.04	0.17	0.47	0.71	0.80	0.60	0.51

Supplementary Table S4. Evolution of water levels at the station My Thuan (Vietnam) over the seasonal cycle 2018. Raw water level data was provided by the SRHMC.

Dune field	Time	Mean dune length $\overline{\lambda}$ [m]	Mean dune height $\overline{\eta}$ [m]	Mean dune asymmetry $\overline{A}$ [-]
DF01	10/02/2018	67.20	1.08	0.42
DF02	05/03/2018	30.24	0.57	0.42

Supplementary Table S5. Characteristic parameters of primary dunes within dune fields *DF01* (wet season) and *DF02* (dry season). Parameters for *DF01* were calculated based on transect E-F (see Fig. 3c) for October 2, 2018. Parameters for *DF02* were calculated based on transect C-D (see Fig. 3a) for May 3, 2018. The bedform tracking tool[13] was used to analyse the primary dunes within datasets.

References

1. Lesser, G. R., Roelvink, J. A., van Kester, J. A. T. M. & Stelling, G. S. Development and validation of a three-dimensional morphological model. *Coast. Eng.* **51**, 883–915, DOI: 10.1016/j.coastaleng.2004.07.014 (2004).
2. Deltares. *Delft3D-FLOW - User Manual, Simulation of multi-dimensional hydrodynamic flows and transport phenomena, including sediments* (Deltares, Delft, The Netherlands, 2019).
3. Wessel, B. *et al.* Accuracy assessment of the global TanDEM-X Digital Elevation Model with GPS data. *ISPRS J. Photogramm. Remote. Sens.* **139**, 171–182, DOI: 10.1016/j.isprsjprs.2018.02.017 (2018).
4. van Rijn, L. C. Unified View of Sediment Transport by Currents and Waves. I: Initiation of Motion, Bed Roughness, and Bed-Load Transport. *J. Hydraul. Eng.* **133**, 649–667, DOI: 10.1061/(ASCE)0733-9429(2007)133:6(649) (2007).
5. Vinh, V. D., Ouillon, S., Thao, N. V. & Tien, N. N. Numerical Simulations of Suspended Sediment Dynamics Due to Seasonal Forcing in the Mekong Coastal Area. *Water* **8**, 255, DOI: 10.3390/w8060255 (2016).
6. Thanh, V. Q., Reyns, J., Wackerman, C., Eidam, E. F. & Roelvink, D. Modelling suspended sediment dynamics on the subaqueous delta of the Mekong River. *Cont. Shelf Res.* **147**, 213–230, DOI: 10.1016/j.csr.2017.07.013 (2017).
7. Gratiot, N. *et al.* Sediment flocculation in the Mekong River estuary, Vietnam, an important driver of geomorphological changes. *Comptes Rendus Geosci.* **349**, 260–268, DOI: 10.1016/j.crte.2017.09.012 (2017).
8. van Rijn, L. C. *Mathematical Modelling of Morphological Processes in the case of Suspended Sediment Transport* (Delft Technical University, Delft, The Netherlands, 1987).
9. Manh, N. V., Dung, N. V., Hung, N. N., Merz, B. & Apel, H. Large-scale suspended sediment transport and sediment deposition in the Mekong Delta. *Hydrol. Earth Syst. Sci.* **18**, 3033–3053, DOI: 10.5194/hess-18-3033-2014 (2014).
10. van Rijn, L. C. *et al.* Simulation of nearshore hydrodynamics and morphodynamics on the time scale of storms and seasons using process-based profile models. In van Rijn, L. C., Ruessink, B. G. & Mulder, J. P. M. (eds.) *Coast3D - Egmond: the behaviour of a straight sandy coast on the time scale of storms and seasons: process knowledge and guidelines for coastal management: end document, March 2002*, S1–S33 (Aqua Publications, Amsterdam, The Netherlands, 2002).
11. Sutherland, J., Peet, A. H. & Soulsby, R. L. Evaluating the performance of morphological models. *Coast. Eng.* **51**, 917–939, DOI: 10.1016/j.coastaleng.2004.07.015 (2004).
12. Ha, D. T., Ouillon, S. & Vinh, G. V. Water and Suspended Sediment Budgets in the Lower Mekong from High-Frequency Measurements (2009–2016). *Water* **10**, 846, DOI: 10.3390/w10070846 (2018).
13. van der Mark, C. F., Blom, A. & Hulscher, S. J. M. H. Quantification of variability in bedform geometry. *J. Geophys. Res.* **113**, F03020, DOI: 10.1029/2007JF000940 (2008).
